# Supplementary material for: Mental impact of Covid-19 among Spanish healthcare workers. A large longitudinal survey
Source: Epidemiol Psychiatr Sci. 2022 Apr 29;31:e28. doi: 10.1017/S2045796022000130 (PMC9069586; doi:10.1017/S2045796022000130)
Supplement: Supplementary file 1 [file epssup.zip › S2045796022000130sup002.docx]

**Mental Impact of Covid-19 among Spanish Healthcare Workers. A large longitudinal survey.**

**Authors:** Alonso J, et al.

**SUPPLEMENTARY TABLES**

**Supplementary Table 1. Comparison of baseline and 4-month follow-up samples. Spanish healthcare workers, MINDCOVID study (absolute numbers and weighted proportions).**

|  | **Full baseline sample**  **(n= 8,996)** | | **FU sample (n=4,809)** | | **Lost to follow up** | **Follow-up responders** |
| --- | --- | --- | --- | --- | --- | --- |
|  | **n^a^** | **%^b^ (SE)** | **n^a^** | **%^c^ (SE)** | **%^b^ (SE)** | **%^b^ (SE)** |
| Total |  |  |  |  | N=4187  46.5 (2.5) | N=4809  53.5 (2.5) |
| Age - 50 years or more | 3623 | 43.4 (2.7) | 2031 | 43.5 (2.5) | 41.1 (3.0) | 45.4 (2.5) |
| Age - 30-49 years | 4197 | 45.8 (1.5) | 2275 | 45.5 (1.6) | 45.6 (1.8) | 46.1 (1.7) |
| Age - 18-29 years | 1176 | 10.8 (1.7) | 503 | 11.0 (1.9) | 13.3 (2.1) | 8.5 (1.3) |
| *modified Rao-Scott (R-S) Χ^2^ test* |  |  |  |  |  | *9.96 (2,9958214) [<0.001]** |
| Gender - Female | 7263 | 77.3 (1.3) | 3899 | 77.6 (1.3) | 77.3 (1.6) | 77.3 (1.2) |
| Gender - Male | 1733 | 22.7 (1.3) | 910 | 22.4 (1.3) | 22.7 (1.6) | 22.7 (1.2) |
| *modified Rao-Scott (R-S) Χ^2^ test* |  |  |  |  |  | *0.00 (1,1.7551E8) [0.971]* |
| Country of birth - Other | 449 | 4.5 (0.5) | 227 | 4.6 (0.5) | 4.7 (0.7) | 4.4 (0.4) |
| Country of birth - Spain | 8547 | 95.5 (0.5) | 4582 | 95.4 (0.5) | 95.3 (0.7) | 95.6 (0.4) |
| *modified Rao-Scott (R-S) Χ^2^ test* |  |  |  |  |  | *0.29 (1,35355) [0.588]* |
| Marital status - Single | 4392 | 46.9 (2.1) | 2272 | 47.3 (2.2) | 48.3 (2.2) | 45.7 (2.1) |
| Marital status - Married | 4605 | 53.1 (2.1) | 2537 | 52.7 (2.2) | 51.7 (2.2) | 54.3 (2.1) |
| *modified Rao-Scott (R-S) Χ^2^ test* |  |  |  |  |  | *3.99 (1,756533) [0.046]** |
| Income - More than 4 | 2926 | 29.9 (1.4) | 1659 | 28.8 (1.5) | 28.1 (1.7) | 31.5 (1.5) |
| Income - Between 2 | 3077 | 34.3 (1.0) | 1730 | 36.1 (1.2) | 31.7 (1.2) | 36.6 (1.1) |
| Income - Less than 2 | 2993 | 35.7 (2.2) | 1420 | 35.1 (2.2) | 40.2 (2.3) | 31.8 (2.1) |
| *modified Rao-Scott (R-S) Χ^2^ test* |  |  |  |  |  | *16.41 (2,122) [<0.001]** |
| Children in care - Yes | 3602 | 40.9 (1.5) | 1995 | 41.2 (1.4) | 39.6 (2.0) | 42.1 (1.3) |
| Children in care - No | 5395 | 59.1 (1.5) | 2814 | 58.8 (1.4) | 60.4 (2.0) | 57.9 (1.3) |
| *modified Rao-Scott (R-S) Χ^2^ test* |  |  |  |  |  | *2.58 (1,25937) [0.108]* |
| Profession - Medical doctor | 2870 | 26.2 (2.8) | 1650 | 26.3 (2.8) | 23.8 (2.8) | 28.2 (2.9) |
| Profession - Nurse | 2660 | 30.8 (1.0) | 1406 | 31.0 (1.2) | 31.1 (1.1) | 30.5 (1.3) |
| Profession - Auxiliary nurse | 874 | 13.6 (3.2) | 387 | 13.7 (3.2) | 16.0 (3.5) | 11.4 (2.9) |
| Profession - Other profession involved in patient care | 980 | 9.2 (0.8) | 555 | 9.0 (0.8) | 8.1 (0.8) | 10.2 (0.9) |
| Profession - Other profession not involved in patient care | 1611 | 20.3 (2.1) | 812 | 20.0 (2.4) | 21.0 (2.0) | 19.7 (2.4) |
| *modified Rao-Scott (R-S) Χ^2^ test* |  |  |  |  |  | *9.40 (4,21979) [<0.001]** |
| Workplace - Hospital | 5457 | 57.6 (13.6) | 2818 | 57.5 (13.5) | 60.3 (12.9) | 55.1 (14.2) |
| Workplace - Primary Care | 2737 | 35.6 (14.3) | 1581 | 36.2 (14.2) | 32.1 (13.6) | 38.6 (14.9) |
| Workplace - Others | 802 | 6.9 (1.3) | 410 | 6.3 (1.3) | 7.6 (1.3) | 6.2 (1.3) |
| *modified Rao-Scott (R-S) Χ^2^ test* |  |  |  |  |  | *4.51 (2,422446) [0.011]** |
| Pre-pandemic mood disorder - Yes | 998 | 11.3 (0.5) | 528 | 11.4 (0.6) | 11.1 (0.8) | 11.4 (0.7) |
| Pre-pandemic mood disorder - No | 7998 | 88.7 (0.5) | 4281 | 88.6 (0.6) | 88.9 (0.8) | 88.6 (0.7) |
| *modified Rao-Scott (R-S) Χ^2^ test* |  |  |  |  |  | *0.06 (1,74322) [0.806]* |
| Pre-pandemic anxiety disorder - Yes | 3211 | 36.1 (0.8) | 1710 | 36.8 (0.9) | 36.3 (0.9) | 36.0 (0.8) |
| Pre-pandemic anxiety disorder - No | 5786 | 63.9 (0.8) | 3099 | 63.2 (0.9) | 63.7 (0.9) | 64.0 (0.8) |
| *modified Rao-Scott (R-S) Χ^2^ test* |  |  |  |  |  | *0.19 (1,1348) [0.663]* |
| Pre-pandemic substance use disorder - Yes | 123 | 1.4 (0.1) | 60 | 1.5 (0.1) | 1.3 (0.2) | 1.4 (0.1) |
| Pre-pandemic substance use disorder - No | 8873 | 98.6 (0.1) | 4749 | 98.5 (0.1) | 98.7 (0.2) | 98.6 (0.1) |
| *modified Rao-Scott (R-S) Χ^2^ test* |  |  |  |  |  | *0.28 (1,262806) [0.598]* |
| Other pre-pandemic mental disorder – Yes | 60 | 0.6 (0.2) | 38 | 0.8 (0.1) | 0.6 (0.2) | 0.7 (0.1) |
| Other pre-pandemic mental disorder - No | 8936 | 99.4 (0.2) | 4772 | 99.2 (0.1) | 99.4 (0.2) | 99.3 (0.1) |
| *modified Rao-Scott (R-S) Χ^2^ test* |  |  |  |  |  | *0.70 (1,3989) [0.404]* |
| Number of pre-pandemic lifetime mental disorders - Two or more | 716 | 8.1 (0.4) | 377 | 8.4 (0.7) | 8.0 (0.7) | 8.2 (0.8) |
| Number of pre-pandemic lifetime mental disorders - Exactly one | 2900 | 32.4 (0.7) | 1550 | 32.8 (0.9) | 32.7 (1.1) | 32.2 (0.8) |
| Number of pre-pandemic lifetime mental disorders - None | 5380 | 59.5 (0.8) | 2882 | 58.8 (0.9) | 59.3 (0.9) | 59.6 (0.8) |
| *modified Rao-Scott (R-S) Χ^2^ test* |  |  |  |  |  | *0.08 (2,267697) [0.922]* |
| Number of physical health conditions - Two or more | 292 | 3.8 (0.4) | 157 | 3.9 (0.5) | 3.6 (0.4) | 4.0 (0.4) |
| Number of physical health conditions - Exactly one | 1872 | 21.7 (0.7) | 1013 | 22.0 (0.8) | 21.2 (1.0) | 22.1 (0.8) |
| Number of physical health conditions - None | 6832 | 74.5 (0.8) | 3639 | 74.1 (1.0) | 75.3 (1.1) | 73.9 (1.0) |
| *modified Rao-Scott (R-S) Χ^2^ test* |  |  |  |  |  | *0.78 (2,228) [0.459]* |
| COVID-19 infection - having been hospitalized for COVID-19 | 109 | 1.2 (0.2) | 67 | 1.4 (0.2) | 1.0 (0.2) | 1.4 (0.2) |
| COVID-19 infection - positive COVID-19 test or medical COVID-19 diagnosis | 1539 | 16.1 (2.1) | 869 | 16.1 (2.0) | 15.0 (2.1) | 17.2 (2.1) |
| COVID-19 infection - none of the above | 7348 | 82.7 (2.2) | 3873 | 82.5 (2.1) | 84.0 (2.3) | 81.5 (2.3) |
| *modified Rao-Scott (R-S) Χ^2^ test* |  |  |  |  |  | *4.14 (2,49270) [0.016]** |
| COVID-19 infection - Type of loved ones infected with COVID-19 - partner, children, or parents | 1371 | 13.9 (2.1) | 781 | 13.7 (2.0) | 12.7 (2.2) | 14.9 (2.1) |
| COVID-19 infection - Type of loved ones infected with COVID-19 - other family, friends or others | 5453 | 58.5 (1.0) | 2964 | 58.6 (0.9) | 56.7 (1.7) | 60.1 (0.8) |
| COVID-19 infection - Type of loved ones infected with COVID-19 - none of the above | 2172 | 27.6 (2.8) | 1065 | 27.7 (2.4) | 30.6 (3.4) | 25.1 (2.4) |
| *modified Rao-Scott (R-S) Χ^2^ test* |  |  |  |  |  | *10.12 (2,510047) [<0.001]** |
| COVID-19 infection - Having been isolated or quarantined because of COVID-19 | 2397 | 25.4 (1.8) | 1337 | 25.6 (1.7) | 24.4 (2.1) | 26.3 (1.7) |
| COVID-19 infection - Not having been isolated or quarantined because of COVID-19 | 6599 | 74.6 (1.8) | 3472 | 74.4 (1.7) | 75.6 (2.1) | 73.7 (1.7) |
| *modified Rao-Scott (R-S) Χ^2^ test* |  |  |  |  |  | *2.42 (1,396699) [0.120]* |
| Work - Frequency of direct exposure to COVID-19 patients 4 | 2209 | 22.9 (3.2) | 1131 | 22.5 (3.2) | 24.3 (3.3) | 21.6 (3.2) |
| Work - Frequency of direct exposure to COVID-19 patients 3 | 1891 | 20.7 (2.0) | 1028 | 20.7 (2.4) | 20.5 (1.7) | 20.9 (2.4) |
| Work - Frequency of direct exposure to COVID-19 patients 2 | 2566 | 30.2 (2.7) | 1407 | 31.5 (2.8) | 28.1 (2.4) | 32.0 (2.9) |
| Work - Frequency of direct exposure to COVID-19 patients 1 | 1168 | 14.1 (1.5) | 623 | 13.5 (1.5) | 14.6 (1.6) | 13.8 (1.5) |
| Work - Frequency of direct exposure to COVID-19 patients 0 | 1162 | 12.0 (2.1) | 620 | 11.8 (2.0) | 12.5 (2.3) | 11.7 (1.9) |
| *modified Rao-Scott (R-S) Χ^2^ test* |  |  |  |  |  | *3.31 (4,3669) [0.010]** |
| Work - Perceived lack of preparedness 4 | 845 | 9.0 (1.1) | 468 | 9.7 (1.4) | 8.7 (1.0) | 9.3 (1.4) |
| Work - Perceived lack of preparedness 3 | 2339 | 25.3 (2.0) | 1257 | 25.5 (2.3) | 25.0 (2.0) | 25.5 (2.2) |
| Work - Perceived lack of preparedness 2 | 3302 | 35.7 (0.7) | 1724 | 34.2 (0.8) | 37.4 (0.9) | 34.3 (0.8) |
| Work - Perceived lack of preparedness 1 | 1842 | 21.3 (2.1) | 1008 | 22.1 (2.5) | 20.0 (1.7) | 22.5 (2.6) |
| Work - Perceived lack of preparedness 0 | 669 | 8.6 (1.4) | 352 | 8.4 (1.3) | 8.8 (1.6) | 8.5 (1.4) |
| *modified Rao-Scott (R-S) Χ^2^ test* |  |  |  |  |  | *1.96 (4,9950) [0.098]* |
| Work - Average weekly hours worked - 51 hours or more | 1457 | 15.2 (0.9) | 765 | 14.0 (0.7) | 15.8 (1.5) | 14.6 (0.7) |
| Work - Average weekly hours worked - 41-50 hours | 2102 | 21.5 (2.1) | 1140 | 22.7 (2.4) | 20.6 (2.2) | 22.3 (2.2) |
| Work - Average weekly hours worked - 40 hours or less | 5437 | 63.3 (2.1) | 2905 | 63.2 (2.5) | 63.6 (2.1) | 63.1 (2.3) |
| *modified Rao-Scott (R-S) Χ^2^ test* |  |  |  |  |  | *1.12 (2,3465) [0.327]* |
| Work - changed to specific COVID-19 related work location | 2108 | 20.7 (3.5) | 1080 | 21.1 (3.7) | 22.1 (3.6) | 19.6 (3.6) |
| Work - changed of team or assigned functions | 3019 | 34.0 (3.2) | 1642 | 33.7 (3.2) | 33.2 (3.3) | 34.8 (3.2) |
| Work - no changes | 3869 | 45.2 (1.5) | 2088 | 45.3 (1.6) | 44.7 (1.7) | 45.7 (1.5) |
| *modified Rao-Scott (R-S) Χ^2^ test* |  |  |  |  |  | *3.92 (2,6661) [0.020]** |
| Work - Perceived frequency of lack of protective equipment 4 | 1120 | 12.5 (1.0) | 558 | 12.5 (1.1) | 13.2 (1.3) | 12.0 (1.0) |
| Work - Perceived frequency of lack of protective equipment 3 | 2336 | 25.5 (1.6) | 1232 | 26.1 (1.6) | 25.7 (1.7) | 25.4 (1.7) |
| Work - Perceived frequency of lack of protective equipment 2 | 3315 | 36.7 (1.1) | 1813 | 37.2 (1.4) | 35.8 (1.1) | 37.5 (1.5) |
| Work - Perceived frequency of lack of protective equipment 1 | 1243 | 14.4 (1.0) | 689 | 13.8 (0.9) | 14.3 (1.4) | 14.5 (0.9) |
| Work - Perceived frequency of lack of protective equipment 0 | 982 | 10.8 (1.3) | 517 | 10.4 (1.2) | 11.0 (1.5) | 10.6 (1.3) |
| *modified Rao-Scott (R-S) Χ^2^ test* |  |  |  |  |  | *0.57 (4,2807) [0.684]* |
| Work - Having to make decisions regarding prioritizing care among COVID-19 patients | 1626 | 16.4 (1.6) | 890 | 15.8 (1.8) | 16.2 (1.6) | 16.5 (1.8) |
| Work - Not having to make decisions regarding prioritizing care among COVID-19 patients | 7370 | 83.6 (1.6) | 3919 | 84.2 (1.8) | 83.8 (1.6) | 83.5 (1.8) |
| *modified Rao-Scott (R-S) Χ^2^ test* |  |  |  |  |  | *0.15 (1,3635) [0.696]* |
| Work - Having patient(s) in care that died from COVID-19 infection | 3530 | 36.9 (3.0) | 1927 | 37.2 (3.2) | 36.4 (2.9) | 37.3 (3.2) |
| Work - Not having patient(s) in care that died from COVID-19 infection | 5466 | 63.1 (3.0) | 2882 | 62.8 (3.2) | 63.6 (2.9) | 62.7 (3.2) |
| *modified Rao-Scott (R-S) Χ^2^ test* |  |  |  |  |  | *0.38 (1,34171) [0.540]* |
| Personal health-related stress (sum G1_1 & G1_6 rescaled to 0-4) 4 | 833 | 9.8 (1.0) | 415 | 10.0 (1.0) | 10.3 (1.1) | 9.4 (0.9) |
| Personal health-related stress (sum G1_1 & G1_6 rescaled to 0-4) 3 | 2158 | 24.3 (0.9) | 1097 | 24.4 (0.9) | 25.8 (1.3) | 23.0 (0.8) |
| Personal health-related stress (sum G1_1 & G1_6 rescaled to 0-4) 2 | 3159 | 34.8 (0.8) | 1680 | 34.7 (0.9) | 34.8 (1.0) | 34.8 (0.8) |
| Personal health-related stress (sum G1_1 & G1_6 rescaled to 0-4) 1 | 2395 | 25.5 (1.1) | 1355 | 25.5 (1.1) | 24.1 (1.4) | 26.8 (1.2) |
| Personal health-related stress (sum G1_1 & G1_6 rescaled to 0-4) 0 | 451 | 5.5 (0.6) | 261 | 5.4 (0.7) | 5.0 (0.6) | 6.0 (0.8) |
| *modified Rao-Scott (R-S) Χ^2^ test* |  |  |  |  |  | *4.04 (4,21334) [0.003]** |
| Health-related stress loved ones (sum G1_2 & G1_7 rescaled to 0-4) 4 | 2875 | 31.8 (2.0) | 1511 | 32.9 (2.2) | 31.7 (2.0) | 31.8 (2.1) |
| Health-related stress loved ones (sum G1_2 & G1_7 rescaled to 0-4) 3 | 2835 | 31.2 (0.5) | 1423 | 29.3 (0.6) | 33.9 (0.8) | 28.9 (0.6) |
| Health-related stress loved ones (sum G1_2 & G1_7 rescaled to 0-4) 2 | 2435 | 26.7 (1.2) | 1392 | 27.5 (1.2) | 24.7 (1.4) | 28.3 (1.2) |
| Health-related stress loved ones (sum G1_2 & G1_7 rescaled to 0-4) 1 | 778 | 9.3 (1.1) | 443 | 9.2 (1.0) | 8.8 (1.1) | 9.8 (1.1) |
| Health-related stress loved ones (sum G1_2 & G1_7 rescaled to 0-4) 0 | 72 | 1.0 (0.2) | 40 | 1.0 (0.2) | 0.9 (0.3) | 1.1 (0.3) |
| *modified Rao-Scott (R-S) Χ^2^ test* |  |  |  |  |  | *6.46 (4,963) [<0.001]** |
| Significant loss of personal or familial income due to COVID-19 - Yes | 1738 | 19.6 (0.7) | 885 | 20.2 (1.2) | 20.4 (0.7) | 19.0 (1.0) |
| Significant loss of personal or familial income due to COVID-19 - No | 7259 | 80.4 (0.7) | 3924 | 79.8 (1.2) | 79.6 (0.7) | 81.0 (1.0) |
| *modified Rao-Scott (R-S) Χ^2^ test* |  |  |  |  |  | *1.94 (1,102642) [0.163]* |
| Financial stress (sum G1_4 & G1_11 rescaled to 0-4) 4 | 445 | 5.2 (0.5) | 195 | 5.1 (0.5) | 6.3 (0.6) | 4.3 (0.4) |
| Financial stress (sum G1_4 & G1_11 rescaled to 0-4) 3 | 955 | 11.2 (0.6) | 471 | 11.4 (0.7) | 12.1 (0.8) | 10.4 (0.6) |
| Financial stress (sum G1_4 & G1_11 rescaled to 0-4) 2 | 1840 | 21.1 (0.6) | 920 | 21.0 (0.7) | 22.3 (0.7) | 20.1 (0.8) |
| Financial stress (sum G1_4 & G1_11 rescaled to 0-4) 1 | 3038 | 33.1 (0.7) | 1610 | 32.5 (0.8) | 33.3 (0.9) | 32.9 (0.7) |
| Financial stress (sum G1_4 & G1_11 rescaled to 0-4) 0 | 2719 | 29.4 (0.8) | 1613 | 30.0 (0.9) | 26.0 (1.0) | 32.3 (0.9) |
| *modified Rao-Scott (R-S) Χ^2^ test* |  |  |  |  |  | *15.44 (4,582) [<0.001]** |
| Interpersonal stress (sum G1_8 G1_9 G1_10 G1_12 rescaled to 0-4) 4 | 421 | 4.9 (0.4) | 198 | 4.8 (0.4) | 5.4 (0.6) | 4.5 (0.3) |
| Interpersonal stress (sum G1_8 G1_9 G1_10 G1_12 rescaled to 0-4) 3 | 1832 | 21.3 (0.7) | 947 | 21.8 (0.9) | 22.1 (1.0) | 20.7 (0.9) |
| Interpersonal stress (sum G1_8 G1_9 G1_10 G1_12 rescaled to 0-4) 2 | 3034 | 33.1 (1.0) | 1588 | 32.7 (1.3) | 34.1 (0.9) | 32.2 (1.3) |
| Interpersonal stress (sum G1_8 G1_9 G1_10 G1_12 rescaled to 0-4) 1 | 2904 | 31.5 (1.1) | 1613 | 31.3 (1.4) | 30.1 (1.3) | 32.6 (1.5) |
| Interpersonal stress (sum G1_8 G1_9 G1_10 G1_12 rescaled to 0-4) 0 | 806 | 9.2 (0.8) | 463 | 9.4 (0.8) | 8.3 (1.1) | 10.0 (0.9) |
| *modified Rao-Scott (R-S) Χ^2^ test* |  |  |  |  |  | *2.22 (4,8976) [0.065]* |
| Major depression disorder (PHQ-8) - Yes | 2510 | 28.0 (1.8) | 1352 | 28.0 (1.9) | 27.8 (2.1) | 28.1 (1.8) |
| Major depression disorder (PHQ-8) - No | 6486 | 72.0 (1.8) | 3457 | 72.0 (1.9) | 72.2 (2.1) | 71.9 (1.8) |
| *modified Rao-Scott (R-S) Χ2 test* |  |  |  |  |  | 0.08 (1,6572044) [0.774] |
| Generalized anxiety disorder (GAD-7) - Yes | 1974 | 22.4 (1.4) | 1037 | 22.6 (1.6) | 22.6 (1.4) | 22.3 (1.5) |
| Generalized anxiety disorder (GAD-7) - No | 7022 | 77.6 (1.4) | 3772 | 77.4 (1.6) | 77.4 (1.4) | 77.7 (1.5) |
| *modified Rao-Scott (R-S) Χ2 test* |  |  |  |  |  | 0.16 (1,5615154) [0.692] |
| Post-traumatic stress disorder (PTSD PCL-5) - Yes | 1911 | 22.1 (1.3) | 1013 | 22.1 (1.3) | 21.9 (1.5) | 22.3 (1.2) |
| Post-traumatic stress disorder (PTSD PCL-5) - No | 7085 | 77.9 (1.3) | 3796 | 77.9 (1.3) | 78.1 (1.5) | 77.7 (1.2) |
| *modified Rao-Scott (R-S) Χ2 test* |  |  |  |  |  | 0.35 (1,291303) [0.556] |
| Panic attacks - Yes | 2035 | 23.9 (1.2) | 1032 | 23.9 (1.3) | 25.0 (1.5) | 23.0 (1.2) |
| Panic attacks - No | 6961 | 76.1 (1.2) | 3777 | 76.1 (1.3) | 75.0 (1.5) | 77.0 (1.2) |
| *modified Rao-Scott (R-S) Χ2 test* |  |  |  |  |  | 4.20 (1,2256) [0.041]* |
| Substance use disorders (CAGE-AID) - Yes | 562 | 6.2 (0.3) | 289 | 6.1 (0.4) | 6.4 (0.5) | 6.0 (0.4) |
| Substance use disorders (CAGE-AID) - No | 8434 | 93.8 (0.3) | 4520 | 93.9 (0.4) | 93.6 (0.5) | 94.0 (0.4) |
| *modified Rao-Scott (R-S) Χ2 test* |  |  |  |  |  | 0.35 (1,7644) [0.552] |
| Any current mental disorder - Yes | 4057 | 45.7 (1.8) | 2128 | 45.4 (1.8) | 46.6 (2.0) | 45.0 (1.7) |
| Any current mental disorder - No | 4939 | 54.3 (1.8) | 2681 | 54.6 (1.8) | 53.4 (2.0) | 55.0 (1.7) |
| *modified Rao-Scott (R-S) Χ2 test* |  |  |  |  |  | 5.97 (1,13771) [0.015]* |

Abbreviations: SE = standard error; R-S = Rao-Scott; COVID-19 = coronavirus disease 2019.

a Unweighted numbers.

b Weighted percentage, using weights obtained for the full baseline sample, which consists on post-stratification weights through raking to restore distributions from the target population in terms of age, gender, profession, and healthcare center.

^C^ Weighted percentage, using follow up weights, which consists on inverse-probability weighting (IPW) to correct bias caused by lost to follow up and post-stratification weights through raking to restore distributions from the target population in terms of age, gender, profession, and healthcare center.

**Supplementary Table 2: Cross-tabulation of PHQ-8 scores between baseline and 4-months follow-up assessments. Spanish healthcare workers, MINDCOVID study (weighted percentages).**

| **PHQ-8 score severity, baseline** | **PHQ-8 score severity, 4-month follow-up** | | | |
| --- | --- | --- | --- | --- |
|  | **Mild (<10)** | **Moderate 10-14** | **Severe 15+** | **Total** |
| **Mild (<10)** | 63.25 | 6.24 | 2.47 | 71.96 |
| **Moderate 10-14** | 9.72 | 5.34 | 2.43 | 17.49 |
| **Severe 15+** | 2.97 | 3.39 | 4.17 | 10.53 |
| **Total** | 75.94 | 14.97 | 9.07 | 100 |

**Supplementary Table 3: Cross-tabulation of GAD-7 scores between baseline and 4-months follow-up assessments. Spanish healthcare workers, MINCOVID study (weighted percentages).**

| **GAD-7 score severity, Baseline** | **GAD-7 score severity. 4-month follow-up** | | | |
| --- | --- | --- | --- | --- |
|  | **Mild (<10)** | **Moderate 10-14** | **Severe 15+** | **Total** |
| **Mild (<10)** | 68.85 | 6.84 | 1.72 | 77.41 |
| **Moderate 10-14** | 8.71 | 3.53 | 2.06 | 14.30 |
| **Severe 15+** | 2.99 | 2.26 | 3.04 | 8.29 |
| **Total** | 80.55 | 12.63 | 6.82 | 100 |

**Supplementary Table 4: Unadjusted associations between distal risk factors and any probable mental disorder at follow-up. Spanish healthcare workers, MINDCOVID study.**

|  | **Full Follow-up sample**  **(n=4,809)** | | | | **Among those with NO baseline disorders**  **(n=2,681)** | | | | **Among those with any baseline disorder (n=2,128)** | | |
| --- | --- | --- | --- | --- | --- | --- | --- | --- | --- | --- | --- |
|  | | | **Any baseline disorder (n=2,128)** | **Any follow-up isorder (n=1,870)** |  | | **Incidence of any disorder (n=480)** |  | | | **Persistence Any Disorder (n=1,390)** |
|  | **n^a^** | **%^b^ (SE)** | **OR (95% CI)** | **OR (95% CI)** | **n^a^** | **%^b^ (SE)** | **OR (95% CI)** | **n^a^** | | **%^b^ (SE)** | **OR (95% CI)** |
| Age - 50 years or more | 2031 | 43.5 (2.5) | 0.58 (0.44-0.75)* | 0.68 (0.57-0.81)* | 1237 | 48.2 (2.6) | 0.66 (0.43-1.00)* | 795 | | 37.8 (2.7) | 0.99 (0.67-1.47) |
| Age - 30-49 years | 2275 | 45.5 (1.6) | 0.85 (0.60-1.21) | 0.91 (0.78-1.06) | 1223 | 42.4 (2.1) | 0.84 (0.55-1.28) | 1052 | | 49.2 (2.1) | 1.02 (0.75-1.40) |
| Age - 18-29 years | 503 | 11.0 (1.9) | (ref) | (ref) | 222 | 9.3 (1.4) | (ref) | 281 | | 13.0 (2.7) | (ref) |
| Fvalue (ndf,ddf)[Pvalue]^c^ |  |  | 17.56(2,412)[<0.001]* | 12.34(2,3134)[<0.001]* |  |  | 3.87(2,6102)[0.021]* |  | |  | 0.09(2,4628)[0.910] |
| Gender - Female | 3899 | 77.6 (1.3) | 1.35 (1.13-1.61)* | 1.25 (1.07-1.46)* | 2096 | 75.1 (1.6) | 1.29 (0.83-2.01) | 1803 | | 80.7 (1.3) | 0.93 (0.70-1.25) |
| Gender - Male | 910 | 22.4 (1.3) | (ref) | (ref) | 585 | 24.9 (1.6) | (ref) | 325 | | 19.3 (1.3) | (ref) |
| Fvalue (ndf,ddf)[Pvalue]^c^ |  |  | 10.75(1,2313)[0.001]* | 7.55(1,14508)[0.006]* |  |  | 1.28(1,67452)[0.258] |  | |  | 0.21(1,100065)[0.643] |
| Country of birth - Other | 227 | 4.6 (0.5) | 1.29 (0.85-1.95) | 1.36 (1.03-1.80)* | 111 | 4.1 (0.5) | 1.43 (0.77-2.65) | 116 | | 5.2 (0.9) | 1.24 (0.75-2.06) |
| Country of birth - Spain | 4582 | 95.4 (0.5) | (ref) | (ref) | 2570 | 95.9 (0.5) | (ref) | 2012 | | 94.8 (0.9) | (ref) |
| Fvalue (ndf,ddf)[Pvalue]^c^ |  |  | 1.41(1,4760)[0.235] | 4.62(1,42329)[0.032]* |  |  | 1.28(1,36257)[0.257] |  | |  | 0.72(1,4714)[0.397] |
| Marital status - Single, divorced, legally separated, or widowed | 2272 | 47.3 (2.2) | 1.28 (1.09-1.51)* | 1.19 (0.98-1.44) | 1199 | 44.1 (2.0) | 0.96 (0.65-1.42) | 1073 | | 51.0 (2.8) | 1.22 (0.94-1.59) |
| Marital status - Married | 2537 | 52.7 (2.2) | (ref) | (ref) | 1482 | 55.9 (2.0) | (ref) | 1054 | | 49.0 (2.8) | (ref) |
| Fvalue (ndf,ddf)[Pvalue]^c^ |  |  | 8.95(1,10605)[0.003]* | 3.12(1,54726)[0.077] |  |  | 0.04(1,36840)[0.838] |  | |  | 2.28(1,36312)[0.131] |
| Income - More than 4,500€ | 1659 | 28.8 (1.5) | 0.51 (0.41-0.62)* | 0.53 (0.43-0.65)* | 1050 | 32.8 (1.8) | 0.58 (0.39-0.86)* | 609 | | 24.1 (1.6) | 0.72 (0.52-0.99)* |
| Income - Between 2,200€ - 4,500€ | 1730 | 36.1 (1.2) | 0.63 (0.51-0.78)* | 0.73 (0.62-0.86)* | 982 | 37.9 (1.4) | 0.79 (0.54-1.15) | 748 | | 33.9 (1.5) | 0.96 (0.78-1.18) |
| Income - Less than 2,200€ | 1420 | 35.1 (2.2) | (ref) | (ref) | 649 | 29.3 (2.1) | (ref) | 771 | | 42.0 (2.6) | (ref) |
| Fvalue (ndf,ddf)[Pvalue]^c^ |  |  | 15.66(2,83)[<0.001]* | 14.70(2,90)[<0.001]* |  |  | 3.99(2,97)[0.022]* |  | |  | 2.38(2,705)[0.094] |
| Children in care - Yes | 1995 | 41.2 (1.4) | 0.91 (0.71-1.17) | 0.92 (0.76-1.10) | 1139 | 42.5 (1.1) | 1.18 (0.92-1.53) | 856 | | 39.7 (2.7) | 0.76 (0.55-1.05) |
| Children in care - No | 2814 | 58.8 (1.4) | (ref) | (ref) | 1542 | 57.5 (1.1) | (ref) | 1272 | | 60.3 (2.7) | (ref) |
| Fvalue (ndf,ddf)[Pvalue]^c^ |  |  | 0.48(1,35532)[0.486] | 0.87(1,42952)[0.352] |  |  | 1.69(1,9375)[0.194] |  | |  | 2.79(1,40870)[0.095] |
| Profession - Medical doctor | 1650 | 26.3 (2.8) | 0.64 (0.48-0.85)* | 0.64 (0.48-0.84)* | 1041 | 30.6 (3.0) | 0.72 (0.47-1.10) | 608 | | 21.2 (2.8) | 0.78 (0.59-1.02) |
| Profession - Nurse | 1406 | 31.0 (1.2) | 1.10 (0.72-1.66) | 0.94 (0.64-1.39) | 697 | 28.7 (1.2) | 0.85 (0.55-1.32) | 709 | | 33.7 (1.6) | 0.91 (0.61-1.34) |
| Profession - Auxiliary nurse | 387 | 13.7 (3.2) | 1.56 (1.06-2.30)* | 1.50 (1.07-2.10)* | 157 | 10.8 (2.5) | 1.20 (0.73-1.98) | 230 | | 17.1 (4.2) | 1.36 (0.89-2.08) |
| Profession - Other profession involved in patient care | 555 | 9.0 (0.8) | 0.79 (0.49-1.26) | 0.80 (0.48-1.34) | 324 | 9.7 (0.9) | 0.75 (0.40-1.41) | 231 | | 8.1 (1.1) | 1.01 (0.70-1.44) |
| Profession - Other profession not involved in patient care | 812 | 20.0 (2.4) | (ref) | (ref) | 462 | 20.1 (1.9) | (ref) | 350 | | 19.9 (3.7) | (ref) |
| Fvalue (ndf,ddf)[Pvalue]^c^ |  |  | 20.71(4,268)[<0.001]* | 9.81(4,85)[<0.001]* |  |  | 4.45(4,214)[0.002]* |  | |  | 3.30(4,230)[0.012]* |
| Workplace - Others | 410 | 6.3 (0.4) | 0.65 (0.46-0.92)* | 0.76 (0.55-1.05) | 258 | 7.3 (0.7) | 0.88 (0.60-1.30) | 153 | | 5.1 (0.6) | 0.97 (0.60-1.55) |
| Workplace - Primary Care | 1581 | 36.2 (0.9) | 0.89 (0.71-1.11) | 1.12 (0.89-1.40) | 899 | 37.0 (1.2) | 1.26 (0.96-1.67) | 682 | | 35.2 (1.3) | 1.23 (0.84-1.80) |
| Workplace - Hospital | 2818 | 57.5 (0.9) | (ref) | (ref) | 1525 | 55.7 (1.2) | (ref) | 1293 | | 59.7 (1.4) | (ref) |
| Fvalue (ndf,ddf)[Pvalue]^c^ |  |  | 3.02(2,147032)[0.049]* | 3.81(2,1053)[0.023]* |  |  | 2.05(2,2131)[0.129] |  | |  | 0.59(2,33505)[0.555] |
| Pre-pandemic mood disorder - Yes | 528 | 11.4 (0.6) | 3.20 (2.50-4.09)* | 2.86 (2.04-4.01)* | 164 | 6.2 (0.5) | 2.14 (1.14-4.01)* | 364 | | 17.6 (1.1) | 1.83 (1.29-2.60)* |
| Pre-pandemic mood disorder - No | 4281 | 88.6 (0.6) | (ref) | (ref) | 2517 | 93.8 (0.5) | (ref) | 1764 | | 82.4 (1.1) | (ref) |
| Fvalue (ndf,ddf)[Pvalue]^c^ |  |  | 76.21(1,544)[<0.001]* | 36.58(1,8350)[<0.001]* |  |  | 5.57(1,13437)[0.018]* |  | |  | 11.39(1,13140)[0.001]* |
| Pre-pandemic anxiety disorder - Yes | 1710 | 36.8 (0.9) | 2.93 (2.57-3.33)* | 2.37 (1.97-2.86)* | 670 | 25.8 (0.7) | 1.83 (1.28-2.63)* | 1040 | | 50.1 (1.4) | 1.46 (1.14-1.87)* |
| Pre-pandemic anxiety disorder - No | 3099 | 63.2 (0.9) | (ref) | (ref) | 2012 | 74.2 (0.7) | (ref) | 1088 | | 49.9 (1.4) | (ref) |
| Fvalue (ndf,ddf)[Pvalue]^c^ |  |  | 85.05(1,23)[<0.001]* | 74.47(1,862)[<0.001]* |  |  | 10.44(1,2008)[0.001]* |  | |  | 8.67(1,2031)[0.003]* |
| Pre-pandemic substance use disorder - Yes | 60 | 1.5 (0.1) | 9.63 (3.23-28.73)* | 3.04 (1.58-5.87)* | 12 | 0.3 (0.1) | 1.08 (0.34-3.45) | 49 | | 2.9 (0.4) | 1.31 (0.63-2.74) |
| Pre-pandemic substance use disorder - No | 4749 | 98.5 (0.1) | (ref) | (ref) | 2670 | 99.7 (0.1) | (ref) | 2079 | | 97.1 (0.4) | (ref) |
| Fvalue (ndf,ddf)[Pvalue]^c^ |  |  | 16.87(1,269409)[<0.001]* | 11.17(1,1656)[0.001]* |  |  | 0.04(1,13874)[0.848] |  | |  | 0.50(1,1031)[0.479] |
| Other pre-pandemic mental disorder - Yes | 38 | 0.8 (0.1) | 1.72 (0.84-3.54) | 2.19 (0.89-5.39) | 16 | 0.6 (0.2) | 2.87 (0.85-9.69) | 21 | | 1.0 (0.2) | 1.30 (0.52-3.20) |
| Other pre-pandemic mental disorder - No | 4772 | 99.2 (0.1) | (ref) | (ref) | 2665 | 99.4 (0.2) | (ref) | 2107 | | 99.0 (0.2) | (ref) |
| Fvalue (ndf,ddf)[Pvalue]^c^ |  |  | 2.19(1,91331)[0.139] | 2.92(1,312425)[0.087] |  |  | 2.92(1,313824)[0.088] |  | |  | 0.32(1,861508)[0.575] |
| Number of pre-pandemic lifetime mental disorders - Two or more | 377 | 8.4 (0.7) | 6.49 (4.81-8.76)* | 4.03 (2.62-6.22)* | 84 | 3.5 (0.4) | 2.10 (0.83-5.35) | 293 | | 14.3 (1.3) | 1.96 (1.25-3.08)* |
| Number of pre-pandemic lifetime mental disorders - Exactly one | 1550 | 32.8 (0.9) | 2.59 (2.16-3.11)* | 2.38 (1.89-3.00)* | 689 | 25.6 (0.8) | 2.11 (1.52-2.92)* | 861 | | 41.3 (2.0) | 1.48 (1.13-1.94)* |
| Number of pre-pandemic lifetime mental disorders - None | 2882 | 58.8 (0.9) | (ref) | (ref) | 1909 | 70.8 (0.7) | (ref) | 973 | | 44.4 (1.6) | (ref) |
| Fvalue (ndf,ddf)[Pvalue]^c^ |  |  | 44.92(2,25)[<0.001]* | 33.84(2,1213)[<0.001]* |  |  | 14.41(2,584)[<0.001]* |  | |  | 8.99(2,1506)[<0.001]* |
| Number of physical health conditions - Two or more | 157 | 3.9 (0.5) | 2.32 (1.42-3.79)* | 2.27 (1.56-3.32)* | 57 | 2.6 (0.5) | 1.09 (0.59-2.02) | 100 | | 5.5 (0.7) | 2.38 (1.37-4.13)* |
| Number of physical health conditions - Exactly one | 1013 | 22.0 (0.8) | 1.32 (1.09-1.58)* | 1.31 (1.11-1.54)* | 502 | 20.4 (1.1) | 1.28 (1.00-1.63) | 511 | | 23.9 (1.3) | 1.16 (0.96-1.39) |
| Number of physical health conditions - None | 3639 | 74.1 (1.0) | (ref) | (ref) | 2122 | 77.0 (1.2) | (ref) | 1516 | | 70.6 (1.6) | (ref) |
| Fvalue (ndf,ddf)[Pvalue]^c^ |  |  | 9.91(2,4954)[<0.001]* | 9.11(2,1163)[<0.001]* |  |  | 2.11(2,278)[0.123] |  | |  | 6.47(2,1398)[0.002]* |

Abbreviations: OR = odds ratio; CI = confidence interval; COVID-19 = coronavirus disease 2019, ndf = numerator degrees of freedom; ddf = denominator degrees of freedom.

*Statistically significant (α = .05).

a Unweighted numbers.

b Weighted percentage (using post-stratification weights and inverse-probability weighting (IPW)).

c F-test to evaluate joint significance of categorical predictor levels based on multiple imputations.

**Supplementary Table 5: Multivariable associations between distal risk factors and any mental disorder at follow-up. Spanish healthcare workers, MINDCOVID study.**

|  | **Full Follow-up sample**  **(n=4,809)** | | | | **Among those with NO baseline disorders**  **(n=2,681)** | | | **Among those with any baseline disorder (n=2,128)** | | |
| --- | --- | --- | --- | --- | --- | --- | --- | --- | --- | --- |
|  |  | | **Any baseline disorder (n=2,128)** | **Any follow-up disorder (n=1,870)** |  | | **Incidence of any disorder (n=480)** |  | | **Persistence of any disorder (n=1,390)** |
|  | **n^a^** | **%^b^ (SE)** | **OR (95% CI)** | **OR (95% CI)** | **n^a^** | **%^b^ (SE)** | **OR (95% CI)** | **n^a^** | **%^b^ (SE)** | **OR (95% CI)** |
| Age - 50 years or more | 2031 | 43.5 (2.5) | 0.57 (0.43-0.76)* | 0.66 (0.50-0.87)* | 1237 | 48.2 (2.6) | 0.52 (0.34-0.78)* | 795 | 37.8 (2.7) | 1.12 (0.68-1.85) |
| Age - 30-49 years | 2275 | 45.5 (1.6) | 0.94 (0.67-1.32) | 0.97 (0.75-1.24) | 1223 | 42.4 (2.1) | 0.69 (0.47-1.02) | 1052 | 49.2 (2.1) | 1.25 (0.79-1.99) |
| Age - 18-29 years | 503 | 11.0 (1.9) | (ref) | (ref) | 222 | 9.3 (1.4) | (ref) | 281 | 13.0 (2.7) | (ref) |
| Fvalue (ndf,ddf)[Pvalue]^c^ |  |  | 14.83(2,217)[<0.001]* | 13.27(2,133)[<0.001]* |  |  | 5.45(2,807)[0.004]* |  |  | 0.86(2,10370)[0.422] |
| Gender - Female | 3899 | 77.6 (1.3) | 1.19 (0.97-1.47) | 1.11 (0.91-1.35) | 2096 | 75.1 (1.6) | 1.16 (0.74-1.81) | 1803 | 80.7 (1.3) | 0.96 (0.73-1.25) |
| Gender - Male | 910 | 22.4 (1.3) | (ref) | (ref) | 585 | 24.9 (1.6) | (ref) | 325 | 19.3 (1.3) | (ref) |
| Fvalue (ndf,ddf)[Pvalue]^c^ |  |  | 2.73(1,18654)[0.099] | 1.03(1,77203)[0.311] |  |  | 0.42(1,73721)[0.519] |  |  | 0.10(1,59535)[0.754] |
| Country of birth - Other | 227 | 4.6 (0.5) | 1.29 (0.90-1.86) | 1.36 (1.05-1.75)* | 111 | 4.1 (0.5) | 1.51 (0.80-2.84) | 116 | 5.2 (0.9) | 1.13 (0.66-1.92) |
| Country of birth - Spain | 4582 | 95.4 (0.5) | (ref) | (ref) | 2570 | 95.9 (0.5) | (ref) | 2012 | 94.8 (0.9) | (ref) |
| Fvalue (ndf,ddf)[Pvalue]^c^ |  |  | 1.86(1,1750)[0.173] | 5.39(1,37665)[0.020]* |  |  | 1.63(1,34543)[0.201] |  |  | 0.18(1,11735)[0.670] |
| Marital status - Single, divorced, legally separated, or widowed | 2272 | 47.3 (2.2) | 0.94 (0.79-1.11) | 0.87 (0.68-1.11) | 1199 | 44.1 (2.0) | 0.75 (0.51-1.09) | 1073 | 51.0 (2.8) | 1.00 (0.76-1.32) |
| Marital status - Married | 2537 | 52.7 (2.2) | (ref) | (ref) | 1482 | 55.9 (2.0) | (ref) | 1054 | 49.0 (2.8) | (ref) |
| Fvalue (ndf,ddf)[Pvalue]^c^ |  |  | 0.60(1,2578)[0.440] | 1.26(1,119877)[0.262] |  |  | 2.21(1,4857)[0.137] |  |  | 0.02(1,76057)[0.902] |
| Income - More than 4,500€ | 1659 | 28.8 (1.5) | 0.70 (0.54-0.90)* | 0.66 (0.51-0.84)* | 1050 | 32.8 (1.8) | 0.59 (0.39-0.89)* | 609 | 24.1 (1.6) | 0.87 (0.62-1.22) |
| Income - Between 2,200€ - 4,500€ | 1730 | 36.1 (1.2) | 0.68 (0.55-0.85)* | 0.78 (0.65-0.94)* | 982 | 37.9 (1.4) | 0.78 (0.56-1.08) | 748 | 33.9 (1.5) | 1.02 (0.81-1.29) |
| Income - Less than 2,200€ | 1420 | 35.1 (2.2) | (ref) | (ref) | 649 | 29.3 (2.1) | (ref) | 771 | 42.0 (2.6) | (ref) |
| Fvalue (ndf,ddf)[Pvalue]^c^ |  |  | 6.02(2,568)[0.003]* | 5.54(2,659)[0.004]* |  |  | 3.45(2,563)[0.032]* |  |  | 0.57(2,418)[0.566] |
| Children in care - Yes | 1995 | 41.2 (1.4) | 0.93 (0.76-1.14) | 0.91 (0.72-1.14) | 1139 | 42.5 (1.1) | 1.12 (0.87-1.45) | 856 | 39.7 (2.7) | 0.77 (0.52-1.14) |
| Children in care - No | 2814 | 58.8 (1.4) | (ref) | (ref) | 1542 | 57.5 (1.1) | (ref) | 1272 | 60.3 (2.7) | (ref) |
| Fvalue (ndf,ddf)[Pvalue]^c^ |  |  | 0.53(1,25415)[0.465] | 0.70(1,32942)[0.403] |  |  | 0.79(1,2546)[0.375] |  |  | 1.67(1,33284)[0.196] |
| Profession - Medical doctor | 1650 | 26.3 (2.8) | 0.70 (0.51-0.97)* | 0.72 (0.51-1.02) | 1041 | 30.6 (3.0) | 0.77 (0.47-1.28) | 608 | 21.2 (2.8) | 0.87 (0.60-1.27) |
| Profession - Nurse | 1406 | 31.0 (1.2) | 1.19 (0.82-1.72) | 0.98 (0.68-1.42) | 697 | 28.7 (1.2) | 0.83 (0.50-1.36) | 709 | 33.7 (1.6) | 1.01 (0.70-1.46) |
| Profession - Auxiliary nurse | 387 | 13.7 (3.2) | 1.48 (1.02-2.15)* | 1.42 (0.99-2.03) | 157 | 10.8 (2.5) | 1.19 (0.68-2.06) | 230 | 17.1 (4.2) | 1.41 (0.89-2.24) |
| Profession - Other profession involved in patient care | 555 | 9.0 (0.8) | 0.81 (0.51-1.26) | 0.85 (0.51-1.40) | 324 | 9.7 (0.9) | 0.79 (0.42-1.48) | 231 | 8.1 (1.1) | 1.12 (0.80-1.57) |
| Profession - Other profession not involved in patient care | 812 | 20.0 (2.4) | (ref) | (ref) | 462 | 20.1 (1.9) | (ref) | 350 | 19.9 (3.7) | (ref) |
| Fvalue (ndf,ddf)[Pvalue]^c^ |  |  | 12.07(4,142)[<0.001]* | 3.84(4,270)[0.005]* |  |  | 2.14(4,520)[0.075] |  |  | 1.28(4,814)[0.277] |
| Workplace - Others | 410 | 6.3 (0.4) | 0.65 (0.44-0.95)* | 0.75 (0.52-1.09) | 258 | 7.3 (0.7) | 0.81 (0.52-1.27) | 153 | 5.1 (0.6) | 0.97 (0.60-1.55) |
| Workplace - Primary Care | 1581 | 36.2 (0.9) | 1.08 (0.81-1.44) | 1.34 (1.03-1.74)* | 899 | 37.0 (1.2) | 1.40 (1.14-1.73)* | 682 | 35.2 (1.3) | 1.38 (0.93-2.06) |
| Workplace - Hospital | 2818 | 57.5 (0.9) | (ref) | (ref) | 1525 | 55.7 (1.2) | (ref) | 1293 | 59.7 (1.4) | (ref) |
| Fvalue (ndf,ddf)[Pvalue]^c^ |  |  | 3.57(2,24133)[0.028]* | 6.11(2,1117)[0.002]* |  |  | 6.46(2,179)[0.002]* |  |  | 1.35(2,10981)[0.260] |
| Number of pre-pandemic lifetime mental disorders - Two or more | 377 | 8.4 (0.7) | 6.20 (4.58-8.39)* | 3.74 (2.57-5.45)* | 84 | 3.5 (0.4) | 2.08 (0.88-4.91) | 293 | 14.3 (1.3) | 1.90 (1.27-2.85)* |
| Number of pre-pandemic lifetime mental disorders - Exactly one | 1550 | 32.8 (0.9) | 2.50 (2.10-2.98)* | 2.29 (1.87-2.81)* | 689 | 25.6 (0.8) | 2.05 (1.57-2.67)* | 861 | 41.3 (2.0) | 1.45 (1.11-1.88)* |
| Number of pre-pandemic lifetime mental disorders - None | 2882 | 58.8 (0.9) | (ref) | (ref) | 1909 | 70.8 (0.7) | (ref) | 973 | 44.4 (1.6) | (ref) |
| Fvalue (ndf,ddf)[Pvalue]^c^ |  |  | 38.86(2,21)[<0.001]* | 38.83(2,554)[<0.001]* |  |  | 14.33(2,70)[<0.001]* |  |  | 8.21(2,427)[<0.001]* |
| Number of physical health conditions - Two or more | 157 | 3.9 (0.5) | 2.33 (1.34-4.05)* | 2.30 (1.44-3.66)* | 57 | 2.6 (0.5) | 0.97 (0.48-1.96) | 100 | 5.5 (0.7) | 2.52 (1.32-4.82)* |
| Number of physical health conditions - Exactly one | 1013 | 22.0 (0.8) | 1.34 (1.07-1.67)* | 1.31 (1.08-1.59)* | 502 | 20.4 (1.1) | 1.36 (1.06-1.75)* | 511 | 23.9 (1.3) | 1.11 (0.90-1.35) |
| Number of physical health conditions - None | 3639 | 74.1 (1.0) | (ref) | (ref) | 2122 | 77.0 (1.2) | (ref) | 1516 | 70.6 (1.6) | (ref) |
| Fvalue (ndf,ddf)[Pvalue]^c^ |  |  | 6.66(2,4570)[0.001]* | 6.39(2,3972)[0.002]* |  |  | 2.84(2,65)[0.065] |  |  | 4.21(2,5075)[0.015]* |

Abbreviations: OR = odds ratio; CI = confidence interval; ndf = numerator degrees of freedom; ddf = denominator degrees of freedom

Note: Results come from one single model for each outcome variable including all distal risk factors, and week of baseline survey

*Statistically significant (α = .05).

a Unweighted numbers.

b Weighted percentage (using post-stratification weights and inverse-probability weighting (IPW)).

c F-test to evaluate joint significance of categorical predictor levels based on multiple imputations.

.

**Supplementary Table 6: Prevalence, Incidence and Persistence of any mental disorder at follow-up, by proximal risk factors. Spanish healthcare workers, MINDCOVID study (absolute numbers and weighted proportions).**

|  | **Full Follow-up sample**  **(n=4,809)** | | | | **Among those with NO baseline disorders**  **(n=2,681)** | | | **Among those with any baseline disorder (n=2,128)** | | |
| --- | --- | --- | --- | --- | --- | --- | --- | --- | --- | --- |
|  |  | | **Any baseline disorder (n=2,128)** | **Any follow-up disorder (n=1,870)** |  | | **Incidence of any disorder (n=480)** |  | | **Persistence of any disorder (n=1,390)** |
|  | **n^a^** | **%^b^ (SE)** | **%^b^ (SE)** | **%^b^ (SE)** | **n^a^** | **%^b^ (SE)** | **%^b^ (SE)** | **n^a^** | **%^b^ (SE)** | **%^b^ (SE)** |
| Total |  |  | 45.4 (1.8) | 41.5 (1.9) |  |  | 19.7 (1.6) |  |  | 67.7 (2.3) |
| COVID-19 infection - having been hospitalized for COVID-19 | 67 | 1.4 (0.2) | 53.5 (6.4) | 50.3 (7.7) | 32 | 1.2 (0.3) | 19.6 (6.3) | 35 | 1.6 (0.2) | 76.9 (9.0) |
| COVID-19 infection - positive COVID-19 test or medical COVID-19 diagnosis^c^ | 869 | 16.1 (2.0) | 48.6 (2.9) | 47.1 (1.7) | 461 | 15.2 (2.0) | 25.8 (2.3) | 408 | 17.2 (2.1) | 69.6 (2.1) |
| COVID-19 infection - none of the above | 3873 | 82.5 (2.1) | 44.7 (1.7) | 40.2 (1.8) | 2189 | 83.6 (2.0) | 18.5 (1.6) | 1685 | 81.1 (2.3) | 67.1 (2.4) |
| *modified Rao-Scott (R-S) Χ^2^ test* |  |  | *2.78 (2,37380) [0.062]* | *9.24 (2,26944) [<0.001]** |  |  | *8.27 (2,2748) [<0.001]** |  |  | *1.42 (2,107948) [0.241]* |
| COVID-19 infection - Type of loved ones infected with COVID-19 - partner, children, or parents | 781 | 13.7 (2.0) | 52.4 (2.9) | 46.2 (2.3) | 406 | 11.9 (2.2) | 20.5 (1.8) | 375 | 15.8 (1.8) | 69.5 (3.6) |
| COVID-19 infection - Type of loved ones infected with COVID-19 - other family, friends or others^d^ | 2964 | 58.6 (0.9) | 45.9 (1.6) | 41.3 (1.9) | 1637 | 58.1 (1.3) | 19.0 (1.5) | 1327 | 59.2 (1.6) | 67.7 (2.2) |
| COVID-19 infection - Type of loved ones infected with COVID-19 - none of the above | 1065 | 27.7 (2.4) | 40.9 (3.0) | 39.5 (2.1) | 639 | 30.0 (3.0) | 20.7 (2.7) | 426 | 25.0 (2.1) | 66.6 (3.7) |
| *modified Rao-Scott (R-S) Χ^2^ test* |  |  | *5.37 (2,4189) [0.005]** | *3.60 (2,7190) [0.027]** |  |  | *0.45 (2,2479) [0.640]* |  |  | *0.24 (2,10171) [0.788]* |
| COVID-19 isolation - Having been isolated or quarantined because of COVID-19 | 1337 | 25.6 (1.7) | 51.0 (2.2) | 48.8 (2.8) | 671 | 23.0 (1.4) | 24.9 (3.1) | 667 | 28.8 (2.2) | 71.7 (3.4) |
| COVID-19 isolation - Not having been isolated or quarantined because of COVID-19 | 3472 | 74.4 (1.7) | 43.5 (1.9) | 39.0 (1.7) | 2011 | 77.0 (1.4) | 18.1 (1.4) | 1461 | 71.2 (2.2) | 66.1 (2.1) |
| *modified Rao-Scott (R-S) Χ^2^ test* |  |  | *14.51 (1,10257) [<0.001]** | *16.64 (1,37020) [<0.001]** |  |  | *5.73 (1,1599) [0.017]** |  |  | *5.09 (1,2645) [0.024]** |
| Work - Frequency of direct exposure to COVID-19 patients 4 | 1131 | 22.5 (3.2) | 57.1 (3.1) | 51.2 (3.3) | 500 | 17.7 (2.5) | 22.6 (2.7) | 631 | 28.3 (4.1) | 72.7 (3.8) |
| Work - Frequency of direct exposure to COVID-19 patients 3 | 1028 | 20.7 (2.4) | 49.5 (2.1) | 47.1 (3.2) | 532 | 19.1 (2.3) | 26.5 (3.0) | 496 | 22.5 (2.8) | 68.1 (4.2) |
| Work - Frequency of direct exposure to COVID-19 patients 2 | 1407 | 31.5 (2.8) | 44.9 (2.1) | 40.8 (1.8) | 814 | 31.8 (2.9) | 19.3 (3.0) | 593 | 31.1 (3.2) | 67.2 (2.1) |
| Work - Frequency of direct exposure to COVID-19 patients 1 | 623 | 13.5 (1.5) | 34.3 (2.3) | 28.1 (2.3) | 400 | 16.3 (2.1) | 13.6 (1.9) | 222 | 10.2 (1.0) | 56.0 (4.5) |
| Work - Frequency of direct exposure to COVID-19 patients 0 | 620 | 11.8 (2.0) | 30.1 (2.7) | 30.1 (3.7) | 435 | 15.1 (2.2) | 14.8 (2.0) | 186 | 7.9 (1.8) | 65.8 (5.7) |
| *modified Rao-Scott (R-S) Χ^2^ test* |  |  | *13.85 (4,6145) [<0.001]** | *10.07 (4,16371) [<0.001]** |  |  | *4.31 (4,7071) [0.002]** |  |  | *2.32 (4,2545) [0.055]* |
| Work - Perceived lack of preparedness 4 | 468 | 9.7 (1.4) | 67.5 (2.3) | 62.4 (3.9) | 157 | 5.8 (1.0) | 34.0 (4.4) | 311 | 14.4 (1.8) | 76.1 (5.5) |
| Work - Perceived lack of preparedness 3 | 1257 | 25.5 (2.3) | 58.8 (2.3) | 50.7 (1.8) | 555 | 19.2 (2.5) | 23.2 (1.7) | 703 | 33.0 (2.0) | 69.9 (2.6) |
| Work - Perceived lack of preparedness 2 | 1724 | 34.2 (0.8) | 43.2 (2.1) | 40.5 (2.4) | 1011 | 35.6 (1.4) | 19.8 (2.3) | 713 | 32.6 (1.3) | 67.7 (2.4) |
| Work - Perceived lack of preparedness 1 | 1008 | 22.1 (2.5) | 31.3 (2.3) | 29.4 (2.3) | 696 | 27.9 (2.8) | 17.0 (2.9) | 312 | 15.3 (2.1) | 56.4 (3.1) |
| Work - Perceived lack of preparedness 0 | 352 | 8.4 (1.3) | 25.5 (3.6) | 25.4 (4.4) | 262 | 11.5 (1.7) | 12.5 (3.5) | 90 | 4.7 (1.0) | 62.9 (7.7) |
| *modified Rao-Scott (R-S) Χ^2^ test* |  |  | *37.62 (4,902) [<0.001]** | *21.10 (4,10173) [<0.001]** |  |  | *5.05 (4,3065) [<0.001]** |  |  | *3.60 (4,4280) [0.006]** |
| Work - Average weekly hours worked - 51 hours or more | 765 | 14.0 (0.7) | 49.6 (2.4) | 41.6 (2.1) | 397 | 13.0 (0.9) | 14.0 (2.4) | 368 | 15.3 (1.0) | 69.7 (2.4) |
| Work - Average weekly hours worked - 41-50 hours | 1140 | 22.7 (2.4) | 50.2 (3.2) | 45.3 (3.5) | 581 | 20.7 (2.3) | 20.7 (2.4) | 559 | 25.1 (2.9) | 69.7 (4.4) |
| Work - Average weekly hours worked - 40 hours or less | 2905 | 63.2 (2.5) | 42.8 (1.9) | 40.1 (1.9) | 1704 | 66.3 (2.5) | 20.5 (2.3) | 1201 | 59.5 (2.7) | 66.4 (2.2) |
| *modified Rao-Scott (R-S) Χ^2^ test* |  |  | *4.88 (2,79070) [0.008]** | *1.81 (2,46204) [0.163]* |  |  | *1.52 (2,18306) [0.218]* |  |  | *0.80 (2,5303) [0.447]* |
| Work - changed to specific COVID-19 related work location | 1080 | 21.1 (3.7) | 52.1 (2.8) | 45.9 (3.6) | 540 | 18.5 (3.6) | 22.2 (2.8) | 540 | 24.2 (4.0) | 67.7 (4.0) |
| Work - changed of team or assigned functions^e^ | 1642 | 33.7 (3.2) | 48.6 (2.1) | 44.4 (2.0) | 871 | 31.7 (3.3) | 20.9 (2.0) | 770 | 36.0 (3.6) | 69.2 (2.7) |
| Work - no changes | 2088 | 45.3 (1.6) | 39.9 (2.0) | 37.2 (2.0) | 1270 | 49.9 (1.7) | 17.9 (1.8) | 818 | 39.8 (2.0) | 66.3 (2.5) |
| *modified Rao-Scott (R-S) Χ^2^ test* |  |  | *12.66 (2,8538) [<0.001]** | *6.48 (2,64741) [0.002]** |  |  | *1.55 (2,5372) [0.213]* |  |  | *0.44 (2,7632) [0.643]* |
| Work - Perceived frequency of lack of protective equipment 4 | 558 | 12.5 (1.1) | 64.5 (3.4) | 60.0 (4.1) | 196 | 8.1 (1.3) | 28.9 (5.2) | 362 | 17.8 (1.1) | 77.1 (3.2) |
| Work - Perceived frequency of lack of protective equipment 3 | 1232 | 26.1 (1.6) | 56.3 (2.2) | 48.2 (1.6) | 562 | 20.9 (1.9) | 22.7 (2.4) | 670 | 32.4 (1.4) | 68.0 (2.9) |
| Work - Perceived frequency of lack of protective equipment 2 | 1813 | 37.2 (1.4) | 42.9 (2.1) | 39.8 (2.3) | 1070 | 38.9 (2.0) | 20.5 (2.8) | 743 | 35.1 (1.1) | 65.5 (2.4) |
| Work - Perceived frequency of lack of protective equipment 1 | 689 | 13.8 (0.9) | 29.7 (2.7) | 28.2 (2.2) | 479 | 17.8 (1.3) | 14.5 (2.2) | 210 | 9.0 (0.8) | 60.6 (5.3) |
| Work - Perceived frequency of lack of protective equipment 0 | 517 | 10.4 (1.2) | 24.8 (2.2) | 26.1 (2.4) | 375 | 14.3 (1.6) | 14.3 (1.8) | 143 | 5.7 (0.8) | 61.7 (6.0) |
| *modified Rao-Scott (R-S) Χ^2^ test* |  |  | *34.00 (4,937) [<0.001]** | *35.65 (4,3987) [<0.001]** |  |  | *3.88 (4,46239) [0.004]** |  |  | *3.94 (4,10785) [0.003]** |
| Work - Having to make decisions regarding prioritizing care among COVID-19 patients | 890 | 15.8 (1.8) | 49.7 (2.9) | 48.1 (1.9) | 460 | 14.6 (2.1) | 24.6 (2.6) | 430 | 17.3 (1.5) | 71.9 (3.4) |
| Work - Not having to make decisions regarding prioritizing care among COVID-19 patients | 3919 | 84.2 (1.8) | 44.6 (1.8) | 40.2 (1.8) | 2222 | 85.4 (2.1) | 18.8 (1.4) | 1698 | 82.7 (1.5) | 66.8 (2.3) |
| *modified Rao-Scott (R-S) Χ^2^ test* |  |  | *5.01 (1,47625) [0.025]** | *25.70 (1,23361) [<0.001]** |  |  | *5.92 (1,44285) [0.015]** |  |  | *3.32 (1,176738) [0.068]* |
| Work - Having patient(s) in care that died from COVID-19 infection | 1927 | 37.2 (3.2) | 49.3 (2.9) | 44.7 (2.5) | 997 | 34.6 (3.2) | 21.9 (1.9) | 931 | 40.3 (3.7) | 68.2 (3.3) |
| Work - Not having patient(s) in care that died from COVID-19 infection | 2882 | 62.8 (3.2) | 43.1 (1.5) | 39.5 (1.8) | 1685 | 65.4 (3.2) | 18.5 (1.6) | 1197 | 59.7 (3.7) | 67.3 (2.3) |
| *modified Rao-Scott (R-S) Χ^2^ test* |  |  | *4.77 (1,115350) [0.029]** | *4.51 (1,13398) [0.034]** |  |  | *4.33 (1,10024) [0.037]** |  |  | *0.09 (1,16845) [0.766]* |
| Personal health-related stress 4 | 415 | 10.0 (1.0) | 79.9 (2.8) | 70.9 (2.6) | 83 | 3.7 (0.5) | 35.5 (3.2) | 333 | 17.5 (1.5) | 79.8 (3.0) |
| Personal health-related stress 3 | 1097 | 24.4 (0.9) | 62.5 (1.8) | 56.3 (1.8) | 410 | 16.8 (0.9) | 29.6 (3.5) | 687 | 33.6 (1.2) | 72.4 (2.6) |
| Personal health-related stress 2 | 1680 | 34.7 (0.9) | 44.3 (1.8) | 41.2 (2.2) | 949 | 35.3 (1.2) | 23.8 (3.5) | 732 | 33.8 (1.1) | 63.0 (2.9) |
| Personal health-related stress 1 | 1355 | 25.5 (1.1) | 24.4 (1.5) | 22.1 (2.0) | 1016 | 35.3 (1.2) | 11.8 (1.3) | 339 | 13.7 (1.3) | 54.0 (4.3) |
| Personal health-related stress 0 | 261 | 5.4 (0.7) | 10.9 (2.5) | 13.5 (2.4) | 224 | 8.9 (1.2) | 9.0 (2.1) | 37 | 1.3 (0.2) | 50.1 (9.8) |
| *modified Rao-Scott (R-S) Χ^2^ test* |  |  | *68.66 (4,3103) [<0.001]** | *58.90 (4,219) [<0.001]** |  |  | *10.08 (4,1438) [<0.001]** |  |  | *11.43 (4,770) [<0.001]** |
| Health-related stress loved ones 4 | 1511 | 32.9 (2.2) | 68.5 (2.2) | 59.1 (2.1) | 494 | 19.0 (1.8) | 26.5 (1.8) | 1018 | 49.6 (2.2) | 74.1 (2.7) |
| Health-related stress loved ones 3 | 1423 | 29.3 (0.6) | 45.3 (1.8) | 44.1 (2.3) | 786 | 29.4 (1.1) | 26.1 (3.4) | 637 | 29.3 (0.9) | 65.7 (3.0) |
| Health-related stress loved ones 2 | 1392 | 27.5 (1.2) | 30.8 (1.4) | 28.4 (1.7) | 984 | 34.8 (1.2) | 16.0 (1.7) | 408 | 18.7 (1.7) | 56.2 (3.4) |
| Health-related stress loved ones 1 | 443 | 9.2 (1.0) | 11.8 (1.8) | 13.3 (2.4) | 380 | 14.9 (1.5) | 8.5 (2.4) | 63 | 2.4 (0.3) | 48.4 (8.9) |
| Health-related stress loved ones 0 | 40 | 1.0 (0.2) | 2.4 (1.8) | 6.7 (4.0) | 38 | 1.9 (0.4) | 5.9 (4.0) | 2 | 0.1 (0.0) | 36.8 (33.8) |
| *modified Rao-Scott (R-S) Χ^2^ test* |  |  | *69.86 (4,772) [<0.001]** | *54.77 (4,2605) [<0.001]** |  |  | *10.73 (4,7550) [<0.001]** |  |  | *7.32 (4,6697) [<0.001]** |
| Significant loss of personal or familial income due to COVID-19 - Yes | 885 | 20.2 (1.2) | 54.8 (2.9) | 49.2 (2.3) | 417 | 16.7 (1.0) | 24.8 (4.5) | 468 | 24.4 (1.8) | 69.4 (3.3) |
| Significant loss of personal or familial income due to COVID-19 - No | 3924 | 79.8 (1.2) | 43.0 (1.7) | 39.5 (1.9) | 2265 | 83.3 (1.0) | 18.6 (1.4) | 1659 | 75.6 (1.8) | 67.1 (2.3) |
| *modified Rao-Scott (R-S) Χ^2^ test* |  |  | *20.21 (1,14103) [<0.001]** | *21.95 (1,5362) [<0.001]** |  |  | *1.92 (1,38708) [0.165]* |  |  | *0.64 (1,10991) [0.422]* |
| Financial stress 4 | 195 | 5.1 (0.5) | 74.9 (3.9) | 62.9 (3.0) | 56 | 2.4 (0.4) | 37.9 (6.8) | 139 | 8.5 (0.9) | 71.3 (3.5) |
| Financial stress 3 | 471 | 11.4 (0.7) | 60.4 (4.1) | 55.1 (3.5) | 187 | 8.3 (1.0) | 24.2 (3.9) | 283 | 15.2 (1.1) | 75.3 (3.6) |
| Financial stress 2 | 920 | 21.0 (0.7) | 52.9 (2.5) | 50.4 (2.7) | 439 | 18.1 (1.2) | 25.2 (3.0) | 481 | 24.5 (0.9) | 72.8 (2.7) |
| Financial stress 1 | 1610 | 32.5 (0.8) | 43.4 (2.0) | 39.8 (1.9) | 922 | 33.6 (0.9) | 20.1 (2.6) | 689 | 31.1 (1.3) | 65.6 (3.0) |
| Financial stress 0 | 1613 | 30.0 (0.9) | 31.5 (1.9) | 28.2 (1.6) | 1078 | 37.6 (0.9) | 14.5 (1.4) | 536 | 20.8 (1.1) | 57.8 (3.0) |
| *modified Rao-Scott (R-S) Χ^2^ test* |  |  | *33.55 (4,2701) [<0.001]** | *45.15 (4,341) [<0.001]** |  |  | *5.20 (4,3314) [<0.001]** |  |  | *8.52 (4,443) [<0.001]** |
| Interpersonal stress 4 | 198 | 4.8 (0.4) | 80.3 (3.4) | 66.0 (4.1) | 37 | 1.7 (0.3) | 28.6 (8.8) | 161 | 8.4 (0.8) | 75.2 (3.8) |
| Interpersonal stress 3 | 947 | 21.8 (0.9) | 72.2 (1.9) | 64.6 (2.1) | 274 | 11.1 (0.9) | 31.3 (3.2) | 673 | 34.6 (1.3) | 77.4 (2.1) |
| Interpersonal stress 2 | 1588 | 32.7 (1.3) | 50.5 (2.6) | 45.4 (2.3) | 777 | 29.6 (1.3) | 25.9 (2.8) | 810 | 36.3 (1.9) | 64.4 (3.1) |
| Interpersonal stress 1 | 1613 | 31.3 (1.4) | 25.5 (1.3) | 25.2 (1.7) | 1196 | 42.8 (1.4) | 15.6 (1.5) | 417 | 17.6 (1.1) | 53.2 (4.8) |
| Interpersonal stress 0 | 463 | 9.4 (0.8) | 14.4 (2.3) | 16.4 (3.1) | 397 | 14.8 (1.2) | 9.2 (1.7) | 66 | 3.0 (0.5) | 59.6 (7.5) |
| *modified Rao-Scott (R-S) Χ^2^ test* |  |  | *86.09 (4,172) [<0.001]** | *75.19 (4,676) [<0.001]** |  |  | *12.34 (4,2117) [<0.001]** |  |  | *11.02 (4,5081) [<0.001]** |
| Family functioning scale 4 | 12 | 0.3 (0.1) | 88.1 (12.7) | 88.4 (12.6) | 2 | 0.1 (0.1) |  | 11 | 0.5 (0.2) | 97.6 (8.9) |
| Family functioning scale 3 | 156 | 3.6 (0.4) | 74.5 (4.4) | 67.1 (5.9) | 40 | 1.7 (0.3) | 29.7 (10.6) | 116 | 6.0 (0.7) | 79.8 (4.9) |
| Family functioning scale 2 | 788 | 17.5 (1.1) | 59.5 (2.6) | 52.1 (2.8) | 329 | 13.0 (1.1) | 25.5 (5.5) | 459 | 23.0 (1.3) | 70.2 (2.9) |
| Family functioning scale 1 | 2062 | 42.7 (1.0) | 46.3 (1.8) | 41.6 (2.3) | 1115 | 42.0 (1.2) | 19.7 (2.0) | 947 | 43.6 (1.3) | 66.9 (2.8) |
| Family functioning scale 0 | 1791 | 35.9 (1.4) | 34.2 (1.7) | 33.3 (1.8) | 1196 | 43.2 (1.6) | 17.5 (1.3) | 595 | 27.0 (1.3) | 63.6 (3.1) |
| *modified Rao-Scott (R-S) Χ^2^ test* |  |  | *18.72 (4,19) [<0.001]** | *15.74 (4,101) [<0.001]** |  |  | *0.89 (4,16) [0.492]* |  |  | *3.53 (4,56) [0.012]** |
| Parental stress scale 4 | 67 | 1.3 (0.2) | 64.5 (8.5) | 48.1 (11.0) | 23 | 0.9 (0.3) | 31.0 (15.2) | 43 | 1.9 (0.3) | 57.4 (9.8) |
| Parental stress scale 3 | 401 | 8.0 (0.5) | 52.3 (3.7) | 48.6 (2.9) | 187 | 7.0 (0.7) | 24.5 (3.9) | 213 | 9.2 (0.7) | 70.6 (3.8) |
| Parental stress scale 2 | 618 | 12.6 (0.8) | 47.4 (2.6) | 41.7 (2.6) | 345 | 12.1 (0.8) | 19.8 (2.7) | 273 | 13.1 (1.1) | 66.1 (3.4) |
| Parental stress scale 1 | 628 | 13.4 (0.9) | 37.9 (3.7) | 36.4 (2.8) | 391 | 15.3 (1.1) | 21.2 (3.5) | 237 | 11.2 (1.2) | 61.3 (4.1) |
| Parental stress scale 0 | 3095 | 64.6 (1.4) | 45.3 (1.9) | 41.5 (2.1) | 1734 | 64.7 (1.2) | 18.6 (1.5) | 1361 | 64.6 (2.4) | 69.0 (2.8) |
| *modified Rao-Scott (R-S) Χ^2^ test* |  |  | *3.72 (4,229) [0.006]** | *2.08 (4,249) [0.083]* |  |  | *0.86 (4,819) [0.487]* |  |  | *1.42 (4,608) [0.227]* |
|  |  |  |  |  |  |  |  |  |  |  |

Abbreviations: SE = standard error ; COVID-19 = coronavirus disease 2019; R-S = Rao-Scott

*Statistically significant (α = .05).

a Unweighted numbers.

b Weighted percentage (using post-stratification weights and inverse-probability weighting (IPW)).

cThe category “positive COVID‐19 test or medical COVID‐19 diagnosis” excludes those having been hospitalized for COVID‐19.

dThe category “other family, friends, or others” excludes having a partner, children, or parents infected with COVID‐19.

eThe category “changed of team or assigned functions” excludes those that changed to a specific COVID‐19‐related work location.

**
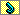
Supplementary Table 7: Multivariable associations of distal risk factors and proximal factors with any probable mental disorder at follow-up. Spanish healthcare workers, MINDCOVID study.**

|  | **Full Follow-up sample**  **(n=4,809)** | | | | **Among those with NO baseline disorders**  **(n=2,681)** | | | **Among those with any baseline disorder (n=2,128)** | | |
| --- | --- | --- | --- | --- | --- | --- | --- | --- | --- | --- |
|  |  | | **Any baseline disorder (n=2,128)** | **Any follow-up disorder (n=1,870)** |  |  | **Incidence of any disorder (n=480)** |  |  | **Persistence of any disorder (n=1,390)** |
|  | **n^a^** | **% (SE) or Med (SE) (IQR)^b^** | **OR (95% CI) ^b^** | **OR (95% CI) ^b^** | **n^a^** | **% (SE) or Med (SE) (IQR)** | **OR (95% CI) ^b^** | **n^a^** | **% (SE) or Med (SE) (IQR) ^b^** | **OR (95% CI) ^b^** |
| Age - 50 years or more | 2031 | 43.5 (2.5) | 0.85 (0.59-1.22) | 0.96 (0.73-1.25) | 1237 | 48.2 (2.6) | 0.68 (0.44-1.06) | 795 | 37.8 (2.7) | 1.35 (0.83-2.18) |
| Age - 30-49 years | 2275 | 45.5 (1.6) | 1.17 (0.81-1.68) | 1.16 (0.92-1.47) | 1223 | 42.4 (2.1) | 0.82 (0.54-1.27) | 1052 | 49.2 (2.1) | 1.37 (0.85-2.20) |
| Age - 18-29 years | 503 | 11.0 (1.9) | (ref) | (ref) | 222 | 9.3 (1.4) | (ref) | 281 | 13.0 (2.7) | (ref) |
| *Fvalue (ndf,ddf)[Pvalue]^c^* |  |  | *3.26(2,3471)[0.039]** | *4.09(2,4822)[0.017]** |  |  | *2.32(2,1241)[0.099]* |  |  | *0.88(2,52505)[0.415]* |
| Gender - Female | 3899 | 77.6 (1.3) | 1.22 (1.00-1.48) | 1.11 (0.87-1.43) | 2096 | 75.1 (1.6) | 1.20 (0.73-1.97) | 1803 | 80.7 (1.3) | 0.98 (0.74-1.29) |
| Gender - Male | 910 | 22.4 (1.3) | (ref) | (ref) | 585 | 24.9 (1.6) | (ref) | 325 | 19.3 (1.3) | (ref) |
| *Fvalue (ndf,ddf)[Pvalue]^c^* |  |  | *3.82(1,6090)[0.051]* | *0.70(1,104766)[0.403]* |  |  | *0.51(1,121687)[0.476]* |  |  | *0.03(1,78504)[0.859]* |
| Country of birth - Other | 227 | 4.6 (0.5) | 1.47 (0.91-2.37) | 1.45 (1.09-1.92)* | 111 | 4.1 (0.5) | 1.42 (0.73-2.78) | 116 | 5.2 (0.9) | 1.27 (0.72-2.22) |
| Country of birth - Spain | 4582 | 95.4 (0.5) | (ref) | (ref) | 2570 | 95.9 (0.5) | (ref) | 2012 | 94.8 (0.9) | (ref) |
| *Fvalue (ndf,ddf)[Pvalue]^c^* |  |  | *2.44(1,1417)[0.118]* | *6.54(1,14926)[0.011]** |  |  | *1.06(1,22055)[0.304]* |  |  | *0.68(1,5540)[0.409]* |
| Marital status - Single, divorced, legally separated, or widowed | 2272 | 47.3 (2.2) | 0.98 (0.79-1.21) | 0.90 (0.67-1.22) | 1199 | 44.1 (2.0) | 0.78 (0.48-1.25) | 1073 | 51.0 (2.8) | 1.02 (0.76-1.37) |
| Marital status - Married | 2537 | 52.7 (2.2) | (ref) | (ref) | 1482 | 55.9 (2.0) | (ref) | 1054 | 49.0 (2.8) | (ref) |
| *Fvalue (ndf,ddf)[Pvalue]^c^* |  |  | *0.04(1,8273)[0.841]* | *0.46(1,213460)[0.499]* |  |  | *1.08(1,18217)[0.300]* |  |  | *0.03(1,36206)[0.874]* |
| Income - More than 4,500€ | 1659 | 28.8 (1.5) | 0.96 (0.73-1.27) | 0.90 (0.70-1.17) | 1050 | 32.8 (1.8) | 0.77 (0.48-1.22) | 609 | 24.1 (1.6) | 1.04 (0.72-1.50) |
| Income - Between 2,200€ - 4,500€ | 1730 | 36.1 (1.2) | 0.81 (0.63-1.03) | 0.95 (0.79-1.15) | 982 | 37.9 (1.4) | 0.93 (0.65-1.33) | 748 | 33.9 (1.5) | 1.11 (0.91-1.37) |
| Income - Less than 2,200€ | 1420 | 35.1 (2.2) | (ref) | (ref) | 649 | 29.3 (2.1) | (ref) | 771 | 42.0 (2.6) | (ref) |
| *Fvalue (ndf,ddf)[Pvalue]^c^* |  |  | *2.38(2,1713)[0.093]* | *0.30(2,1542)[0.738]* |  |  | *0.84(2,406)[0.431]* |  |  | *0.59(2,982)[0.556]* |
| Children in care - Yes | 1995 | 41.2 (1.4) | 0.68 (0.46-1.01) | 0.75 (0.54-1.04) | 1139 | 42.5 (1.1) | 1.02 (0.70-1.47) | 856 | 39.7 (2.7) | 0.66 (0.37-1.17) |
| Children in care - No | 2814 | 58.8 (1.4) | (ref) | (ref) | 1542 | 57.5 (1.1) | (ref) | 1272 | 60.3 (2.7) | (ref) |
| *Fvalue (ndf,ddf)[Pvalue]^c^* |  |  | *3.66(1,3342)[0.056]* | *2.91(1,1241)[0.088]* |  |  | *0.06(1,1094)[0.803]* |  |  | *1.97(1,2649)[0.161]* |
| Profession - Medical doctor | 1650 | 26.3 (2.8) | 0.55 (0.43-0.71)* | 0.54 (0.38-0.77)* | 1041 | 30.6 (3.0) | 0.52 (0.28-0.97)* | 608 | 21.2 (2.8) | 0.76 (0.51-1.15) |
| Profession - Nurse | 1406 | 31.0 (1.2) | 0.79 (0.61-1.02) | 0.62 (0.42-0.92)* | 697 | 28.7 (1.2) | 0.52 (0.28-0.94)* | 709 | 33.7 (1.6) | 0.79 (0.51-1.21) |
| Profession - Auxiliary nurse | 387 | 13.7 (3.2) | 0.92 (0.64-1.31) | 0.88 (0.61-1.27) | 157 | 10.8 (2.5) | 0.77 (0.40-1.48) | 230 | 17.1 (4.2) | 1.12 (0.73-1.72) |
| Profession - Other profession involved in patient care | 555 | 9.0 (0.8) | 0.71 (0.50-1.02) | 0.78 (0.49-1.24) | 324 | 9.7 (0.9) | 0.64 (0.32-1.28) | 231 | 8.1 (1.1) | 1.17 (0.83-1.65) |
| Profession - Other profession not involved in patient care | 812 | 20.0 (2.4) | (ref) | (ref) | 462 | 20.1 (1.9) | (ref) | 350 | 19.9 (3.7) | (ref) |
| *Fvalue (ndf,ddf)[Pvalue]^c^* |  |  | *10.30(4,168)[<0.001]** | *4.57(4,247)[0.001]** |  |  | *3.22(4,1712)[0.012]** |  |  | *2.04(4,666)[0.088]* |
| Workplace - Others | 410 | 6.3 (0.4) | 1.12 (0.87-1.44) | 1.24 (0.85-1.82) | 258 | 7.3 (0.7) | 1.25 (0.81-1.92) | 153 | 5.1 (0.6) | 1.27 (0.75-2.16) |
| Workplace - Primary Care | 1581 | 36.2 (0.9) | 1.06 (0.88-1.28) | 1.33 (1.16-1.51)* | 899 | 37.0 (1.2) | 1.34 (0.98-1.82) | 682 | 35.2 (1.3) | 1.39 (0.97-2.00) |
| Workplace - Hospital | 2818 | 57.5 (0.9) | (ref) | (ref) | 1525 | 55.7 (1.2) | (ref) | 1293 | 59.7 (1.4) | (ref) |
| *Fvalue (ndf,ddf)[Pvalue]^c^* |  |  | *0.53(2,4168)[0.589]* | *9.20(2,991)[<0.001]** |  |  | *2.10(2,940)[0.123]* |  |  | *1.70(2,4551)[0.183]* |
| Number of pre-pandemic lifetime mental disorders - Two or more | 377 | 8.4 (0.7) | 6.03 (4.39-8.28)* | 3.19 (2.16-4.71)* | 84 | 3.5 (0.4) | 2.17 (0.89-5.30) | 293 | 14.3 (1.3) | 1.90 (1.24-2.92)* |
| Number of pre-pandemic lifetime mental disorders - Exactly one | 1550 | 32.8 (0.9) | 2.24 (1.93-2.59)* | 2.01 (1.66-2.43)* | 689 | 25.6 (0.8) | 2.03 (1.54-2.67)* | 861 | 41.3 (2.0) | 1.43 (1.06-1.94)* |
| Number of pre-pandemic lifetime mental disorders - None | 2882 | 58.8 (0.9) | (ref) | (ref) | 1909 | 70.8 (0.7) | (ref) | 973 | 44.4 (1.6) | (ref) |
| *Fvalue (ndf,ddf)[Pvalue]^c^* |  |  | *42.08(2,31)[<0.001]** | *30.40(2,640)[<0.001]** |  |  | *13.51(2,393)[<0.001]** |  |  | *5.68(2,280)[0.004]** |
| Number of physical health conditions - Two or more | 157 | 3.9 (0.5) | 1.95 (1.21-3.15)* | 1.96 (1.33-2.89)* | 57 | 2.6 (0.5) | 0.86 (0.38-1.97) | 100 | 5.5 (0.7) | 2.34 (1.24-4.39)* |
| Number of physical health conditions - Exactly one | 1013 | 22.0 (0.8) | 1.37 (1.08-1.75)* | 1.32 (1.07-1.62)* | 502 | 20.4 (1.1) | 1.45 (1.10-1.90)* | 511 | 23.9 (1.3) | 1.07 (0.86-1.32) |
| Number of physical health conditions - None | 3639 | 74.1 (1.0) | (ref) | (ref) | 2122 | 77.0 (1.2) | (ref) | 1516 | 70.6 (1.6) | (ref) |
| *Fvalue (ndf,ddf)[Pvalue]^c^* |  |  | *8.12(2,1165)[<0.001]** | *5.99(2,2210)[0.003]** |  |  | *3.21(2,93)[0.045]** |  |  | *3.47(2,4998)[0.031]** |
| COVID-19 infection - having been hospitalized for COVID-19 | 67 | 1.4 (0.2) | 0.73 (0.40-1.33) | 0.85 (0.51-1.42) | 32 | 1.2 (0.3) | 1.02 (0.43-2.42) | 35 | 1.6 (0.2) | 0.98 (0.34-2.81) |
| COVID-19 infection - positive COVID-19 test or medical COVID-19 diagnosis | 869 | 16.1 (2.0) | 0.73 (0.58-0.91)* | 0.90 (0.73-1.13) | 461 | 15.2 (2.0) | 1.22 (0.87-1.70) | 408 | 17.2 (2.1) | 0.86 (0.67-1.10) |
| COVID-19 infection - none of the above | 3873 | 82.5 (2.1) | (ref) | (ref) | 2189 | 83.6 (2.0) | (ref) | 1685 | 81.1 (2.3) | (ref) |
| *Fvalue (ndf,ddf)[Pvalue]^c^* |  |  | *3.71(2,671)[0.025]** | *0.68(2,9007)[0.505]* |  |  | *0.66(2,758)[0.516]* |  |  | *0.69(2,848)[0.502]* |
| COVID-19 infection - Type of loved ones infected with COVID-19 - partner, children, or parents | 781 | 13.7 (2.0) | 1.44 (1.13-1.83)* | 1.04 (0.83-1.31) | 406 | 11.9 (2.2) | 0.75 (0.50-1.14) | 375 | 15.8 (1.8) | 1.10 (0.80-1.52) |
| COVID-19 infection - Type of loved ones infected with COVID-19 - other family, friends or others | 2964 | 58.6 (0.9) | 1.12 (0.91-1.37) | 0.98 (0.85-1.13) | 1637 | 58.1 (1.3) | 0.80 (0.63-1.02) | 1327 | 59.2 (1.6) | 1.07 (0.77-1.47) |
| COVID-19 infection - Type of loved ones infected with COVID-19 - none of the above | 1065 | 27.7 (2.4) | (ref) | (ref) | 639 | 30.0 (3.0) | (ref) | 426 | 25.0 (2.1) | (ref) |
| *Fvalue (ndf,ddf)[Pvalue]^c^* |  |  | *4.33(2,1703)[0.013]** | *0.14(2,49046)[0.873]* |  |  | *1.52(2,437)[0.219]* |  |  | *0.18(2,3302)[0.837]* |
| COVID-19 isolation - Having been isolated or quarantined because of COVID-19 | 1337 | 25.6 (1.7) | 1.23 (0.93-1.64) | 1.31 (1.00-1.72) | 671 | 23.0 (1.4) | 1.22 (0.79-1.88) | 667 | 28.8 (2.2) | 1.25 (0.92-1.71) |
| COVID-19 isolation - Not having been isolated or quarantined because of COVID-19 | 3472 | 74.4 (1.7) | (ref) | (ref) | 2011 | 77.0 (1.4) | (ref) | 1461 | 71.2 (2.2) | (ref) |
| *Fvalue (ndf,ddf)[Pvalue]^c^* |  |  | *2.02(1,2341)[0.156]* | *3.83(1,180974)[0.050]* |  |  | *0.81(1,1028)[0.369]* |  |  | *1.99(1,6847)[0.158]* |
| *Work - Frequency of direct exposure to COVID-19 patients* |  | *2.8 (0.2) (2.0-3.9)* | *1.08 (0.98-1.19)* | *1.12 (1.03-1.22)** |  | *2.6 (0.2) (1.6-3.6)* | *1.14 (1.01-1.28)** |  | *3.0 (0.2) (2.2-4.1)* | *1.11 (0.99-1.24)* |
| *Fvalue (ndf,ddf)[Pvalue]c* |  |  | *2.56(1,6489)[0.110]* | *6.37(1,1623)[0.012]** |  |  | *4.70(1,36259)[0.030]** |  |  | *2.82(1,1142)[0.093]* |
| *Work - Perceived lack of preparedness* |  | *1.6 (0.1) (0.7-2.4)* | *1.22 (1.12-1.34)** | *1.17 (1.08-1.27)** |  | *1.3 (0.1) (0.5-2.0)* | *1.16 (1.04-1.29)** |  | *1.9 (0.1) (1.2-2.7)* | *1.09 (0.95-1.26)* |
| *Fvalue (ndf,ddf)[Pvalue]c* |  |  | *19.05(1,2209)[<0.001]** | *14.54(1,3924)[<0.001]** |  |  | *6.13(1,68)[0.016]** |  |  | *1.38(1,3055)[0.240]* |
| Work - Average weekly hours worked - 51 hours or more | 765 | 14.0 (0.7) | 1.43 (1.15-1.79)* | 1.03 (0.83-1.28) | 397 | 13.0 (0.9) | 0.65 (0.39-1.08) | 368 | 15.3 (1.0) | 1.15 (0.92-1.45) |
| Work - Average weekly hours worked - 41-50 hours | 1140 | 22.7 (2.4) | 1.20 (0.97-1.50) | 1.13 (0.89-1.44) | 581 | 20.7 (2.3) | 1.03 (0.73-1.45) | 559 | 25.1 (2.9) | 1.14 (0.78-1.68) |
| Work - Average weekly hours worked - 40 hours or less | 2905 | 63.2 (2.5) | (ref) | (ref) | 1704 | 66.3 (2.5) | (ref) | 1201 | 59.5 (2.7) | (ref) |
| *Fvalue (ndf,ddf)[Pvalue]^c^* |  |  | *6.17(2,1645)[0.002]** | *0.55(2,19516)[0.577]* |  |  | *1.89(2,9339)[0.152]* |  |  | *1.08(2,194)[0.342]* |
| Work - changed to specific COVID-19 related work location | 1080 | 21.1 (3.7) | 1.01 (0.83-1.23) | 1.01 (0.81-1.26) | 540 | 18.5 (3.6) | 1.09 (0.75-1.60) | 540 | 24.2 (4.0) | 0.92 (0.67-1.27) |
| Work - changed of team or assigned functions | 1642 | 33.7 (3.2) | 1.18 (0.97-1.44) | 1.12 (1.00-1.27) | 871 | 31.7 (3.3) | 1.16 (0.89-1.50) | 770 | 36.0 (3.6) | 0.99 (0.84-1.17) |
| Work - no changes | 2088 | 45.3 (1.6) | (ref) | (ref) | 1270 | 49.9 (1.7) | (ref) | 818 | 39.8 (2.0) | (ref) |
| *Fvalue (ndf,ddf)[Pvalue]^c^* |  |  | *1.32(2,1495)[0.268]* | *1.84(2,578)[0.160]* |  |  | *0.57(2,756)[0.566]* |  |  | *0.14(2,6516)[0.869]* |
| *Work - Perceived frequency of lack of protective equipment* |  | *2.7 (0.1) (2.0-3.5)* | *1.14 (1.04-1.26)** | *1.07 (0.98-1.16)* |  | *2.5 (0.1) (1.6-3.2)* | *1.04 (0.90-1.21)* |  | *3.0 (0.1) (2.3-3.8)* | *1.02 (0.91-1.15)* |
| *Fvalue (ndf,ddf)[Pvalue]c* |  |  | *7.59(1,4218)[0.006]** | *2.35(1,38937)[0.126]* |  |  | *0.30(1,64988)[0.581]* |  |  | *0.17(1,31495)[0.685]* |
| Work - Having to make decisions regarding prioritizing care among COVID-19 patients | 890 | 15.8 (1.8) | 1.03 (0.80-1.33) | 1.38 (1.20-1.58)* | 460 | 14.6 (2.1) | 1.32 (1.03-1.68)* | 430 | 17.3 (1.5) | 1.54 (1.29-1.85)* |
| Work - Not having to make decisions regarding prioritizing care among COVID-19 patients | 3919 | 84.2 (1.8) | (ref) | (ref) | 2222 | 85.4 (2.1) | (ref) | 1698 | 82.7 (1.5) | (ref) |
| *Fvalue (ndf,ddf)[Pvalue]^c^* |  |  | *0.05(1,21835)[0.826]* | *18.83(1,373)[<0.001]** |  |  | *4.81(1,6927)[0.028]** |  |  | *21.86(1,989)[<0.001]** |
| Work - Having patient(s) in care that died from COVID-19 infection | 1927 | 37.2 (3.2) | 0.96 (0.84-1.09) | 0.96 (0.79-1.18) | 997 | 34.6 (3.2) | 1.03 (0.77-1.37) | 931 | 40.3 (3.7) | 0.88 (0.66-1.17) |
| Work - Not having patient(s) in care that died from COVID-19 infection | 2882 | 62.8 (3.2) | (ref) | (ref) | 1685 | 65.4 (3.2) | (ref) | 1197 | 59.7 (3.7) | (ref) |
| *Fvalue (ndf,ddf)[Pvalue]^c^* |  |  | *0.46(1,2271)[0.498]* | *0.11(1,3658)[0.744]* |  |  | *0.04(1,15333)[0.848]* |  |  | *0.81(1,4554)[0.369]* |
| *Personal health-related stress (rescaled to 0-4)* |  | *1.6 (0.1) (0.8-2.4)* | *1.37 (1.20-1.55)** | *1.34 (1.20-1.49)** |  | *1.2 (0.0) (0.5-1.9)* | *1.28 (1.10-1.49)** |  | *2.0 (0.1) (1.3-2.8)* | *1.22 (1.07-1.40)** |
| *Fvalue (ndf,ddf)[Pvalue]c* |  |  | *22.35(1,2237)[<0.001]** | *26.68(1,3722)[<0.001]** |  |  | *9.80(1,4241)[0.002]** |  |  | *8.89(1,34620)[0.003]** |
| *Health-related stress loved ones (rescaled to 0-4)* |  | *2.4 (0.1) (1.5-3.2)* | *1.23 (1.08-1.40)** | *1.14 (1.02-1.27)** |  | *2.0 (0.1) (1.2-2.8)* | *1.03 (0.85-1.25)* |  | *3.0 (0.1) (2.1-3.5)* | *1.11 (0.95-1.29)* |
| *Fvalue (ndf,ddf)[Pvalue]c* |  |  | *9.27(1,5312)[0.002]** | *5.04(1,12590)[0.025]** |  |  | *0.11(1,9779)[0.743]* |  |  | *1.63(1,32288)[0.202]* |
| Significant loss of personal or familial income due to COVID-19 - Yes | 885 | 20.2 (1.2) | 1.24 (0.94-1.63) | 1.13 (0.84-1.52) | 417 | 16.7 (1.0) | 1.25 (0.66-2.36) | 468 | 24.4 (1.8) | 0.94 (0.71-1.25) |
| Significant loss of personal or familial income due to COVID-19 - No | 3924 | 79.8 (1.2) | (ref) | (ref) | 2265 | 83.3 (1.0) | (ref) | 1659 | 75.6 (1.8) | (ref) |
| *Fvalue (ndf,ddf)[Pvalue]^c^* |  |  | *2.35(1,9709)[0.125]* | *0.68(1,158173)[0.410]* |  |  | *0.46(1,116347)[0.499]* |  |  | *0.16(1,34296)[0.689]* |
| Financial stress (rescaled to 0-4) |  | 0.6 (0.0) (0.0-1.6) | 0.94 (0.84-1.05) | 1.00 (0.89-1.12) |  | 0.4 (0.0) (0.0-1.2) | 0.96 (0.77-1.18) |  | 0.9 (0.0) (0.1-1.9) | 1.07 (0.93-1.23) |
| *Fvalue (ndf,ddf)[Pvalue]^c^* |  |  | *1.29(1,3520)[0.256]* | *0.01(1,397458)[0.925]* |  |  | *0.17(1,55828)[0.679]* |  |  | *0.82(1,11803)[0.366]* |
| Interpersonal stress score (rescaled to 0-4) |  | 1.3 (0.1) (0.5-2.1) | 1.67 (1.43-1.94)* | 1.41 (1.21-1.64)* |  | 0.8 (0.0) (0.2-1.6) | 1.26 (1.13-1.42)* |  | 1.8 (0.0) (1.1-2.5) | 1.22 (0.99-1.50) |
| *Fvalue (ndf,ddf)[Pvalue]^c^* |  |  | *40.42(1,1207)[<0.001]** | *20.11(1,39682)[<0.001]** |  |  | *16.43(1,1719)[<0.001]** |  |  | *3.40(1,31153)[0.065]* |
| Family functioning scale score (rescaled to 0-4) |  | 0.3 (0.0) (0.0-0.9) | 1.38 (1.24-1.52)* | 1.25 (1.12-1.39)* |  | 0.2 (0.0) (0.0-0.8) | 1.10 (0.92-1.30) |  | 0.5 (0.0) (0.0-1.2) | 1.16 (1.00-1.36) |
| *Fvalue (ndf,ddf)[Pvalue]^c^* |  |  | *19.56(1,30)[<0.001]** | *14.78(1,147)[<0.001]** |  |  | *1.05(1,117)[0.307]* |  |  | *4.04(1,1141)[0.045]** |
| Parental stress scale score (rescaled to 0-4) |  | 0.0 (0.1) (0.0-0.8) | 1.08 (0.90-1.30) | 1.03 (0.88-1.21) |  | 0.0 (0.0) (0.0-0.7) | 1.00 (0.85-1.17) |  | 0.0 (0.1) (0.0-0.9) | 1.04 (0.83-1.30) |
| *Fvalue (ndf,ddf)[Pvalue]^c^* |  |  | *0.72(1,2300)[0.396]* | *0.14(1,4096)[0.708]* |  |  | *0.10(1,1226)[0.755]* |  |  | *0.10(1,4208)[0.747]* |

Abbreviations: OR = odds ratio; CI = confidence interval; ndf = numerator degrees of freedom; ddf = denominator degrees of freedom

Note: Results come from one single model for each outcome variable including all distal and proximal risk factors, and week of baseline survey

*Statistically significant (α = .05).

a Unweighted numbers.

b Weighted percentage (using post-stratification weights and inverse-probability weighting (IPW)).

c F-test to evaluate joint significance of categorical predictor levels based on multiple imputations.
